# Proteomic and Molecular Biological Analyses of Response Mechanism in Rice (*Oryza sativa* L. cv. Koshihikari) Under Drought Stress

**DOI:** 10.3390/ijms27198895

**Published:** 2026-10-07

**Authors:** Sheikh Shohag, Hisateru Yamaguchi, Keisuke Hitachi, Kunihiro Tsuchida, Setsuko Komatsu

**Affiliations:** 1 Department of Applied Sciences and Engineering, Fukui University of Technology, Fukui 910-8505, Japan; 2 Department of Management Nutrition, Aichi Gakusen University, Okazaki 444-8520, Japan; hyamaguchi@gakusen.ac.jp; 3 Center for Medical Science, Fujita Health University, Toyoake 470-1192, Japantsuchida@fujita-hu.ac.jp (K.T.); 4 Faculty of Environment and Information Sciences, Fukui University of Technology, Fukui 910-8505, Japan

**Keywords:** rice, drought stress, polyethylene glycol, proteomics

## Abstract

Drought stress severely limits rice growth, while roots play a key role in sensing water deficits and triggering adaptive responses. To investigate time-dependent molecular responses in rice (*Oryza sativa* L. cv. Koshihikari) under drought stress induced by polyethylene glycol (PEG), seedlings were exposed to 10 and 20% PEG for 4 and 8 days, followed by morphological, proteomic, immunoblot, polymerase chain reaction, and ATP content analyses. Primary root length and total root fresh weight were significantly decreased with 20% PEG for 8 days compared with untreated. Proteomic analysis revealed changes in the abundance of rice root proteins under 20% PEG treatment between 4 and 8 days, with differentially abundant proteins enriched in Gene Ontology categories related to nuclear components, protein phosphorylation, and ATP binding. Selected proteins related to protein protection, redox-associated processes, and cytoskeletal responses were examined using immunoblot and polymerase chain reaction analyses. Glutathione S-transferase decreased and heat shock protein 70 increased with PEG for 8 days. The expression of *NADPH-dependent codeinone reductase* was upregulated and *heat shock protein 17.9* was downregulated by PEG for 8 days. ATP content per unit fresh weight decreased in roots after PEG treatment. These results suggest that prolonged PEG-induced osmotic stress inhibits rice root growth through alterations in cellular energy status, the abundance of proteins involved in protein protection and glutathione S-transferase-mediated detoxification, and the expression of genes associated with reactive carbonyl species metabolism.

## 1. Introduction

Rice is the most widely consumed grain as the primary food source by people globally, especially in Asian nations [1]. Rice accounts for providing 20% of the nutritional energy supply of the world [2]. Rice, which is rich in carbohydrates, quality protein, and micronutrients, is an important part of the daily diet [3]. Climate change is expected to increase the frequency and severity of drought conditions, while pressure on freshwater resources challenges the sustainability of water-intensive rice cultivation [4]. However, as the population continues to rise, the demand for rice production is increasing [5]. Drought stress restricts rice growth by reducing plant height, tillering, leaf development, biomass accumulation, and grain yield [6]. Over one third of the cultivated land of the world is affected by drought stress [7]. Drought stress is a water deficit condition due to insufficient rainfall or higher evaporation rates [8]. It causes impaired rice growth and development by disrupting morpho-physiological, biochemical, and molecular responses [9]. Drought stress during the reproductive stage of rice is particularly severe, as it significantly reduces grain yield [10]. The seedling stage of rice is highly sensitive to water availability, as plants undergo rapid growth during this period and establish roots and shoots, which ultimately influences subsequent growth and grain yield [11]. Therefore, understanding the mechanisms underlying drought responses during the rice seedling stage is crucial for sustaining rice production and ensuring food security, because water deficit at this stage adversely affects subsequent growth and productivity.

Drought stress is initially detected at the roots and transferred to the aerial parts of the plant through sophisticated signaling mechanisms [12]. Drought stress mainly alters water balance in plants, affecting both the aerial and root systems of rice [6]. Plants store osmoprotectants such as proline, glycine, betaine, and sugars to preserve cellular osmotic balance and prevent turgor loss [13]. Two drought-tolerant rice cultivars, 303B and HY796 (*Oryza sativa* L.; *indica* type), exhibited 30–50% higher activities of antioxidant enzymes, including catalase, peroxidase, and superoxide dismutase, and lower malondialdehyde levels, for better protection of cell membranes [14]. *OsDUF1223* regulated drought tolerance at the seedling stage in rice (*O. sativa* L.; *indica*-type) through its functional association with abscisic acid (ABA) and nitrogen signaling pathways, while knockout lines exhibited higher ABA content and improved drought tolerance [15]. Quantitative proteomic analysis of root tips from drought-tolerant genotype Azucena (*O. sativa* L.; *japonica*-type) and drought-sensitive genotype IR64 (*O. sativa* L.; *indica*-type) revealed genotype-dependent changes in proteins involved in root elongation, antioxidant defense, and iron homeostasis [16]. Label-free proteomics of eight rice genotypes under drought identified increased proteins related to redox regulation, protein protection, cytoskeletal remodeling, and mitochondrial function in rice [17]. Collectively, these studies indicate that rice adapts to drought through coordinated changes in osmotic adjustment, antioxidant defense, hormone signaling, and root proteome reprogramming. However, how these responses integrated at the protein level across different genotypes and tissues are still not fully understood.

Polyethylene glycol (PEG) is a nonionic and non-toxic polymer, which can alter the osmotic potential of nutrient medium by lowering water potential without directly damaging plant cells [18]. High molecular weight PEG is a suitable water potential regulator for simulating soil drought [19]. PEG-induced drought stress altered proteins in soybean roots [20] and pitaya stems [21] involved in metabolic pathways. Although transcriptomic and metabolomic studies have provided important insights into rice responses to water deficit, transcript abundance does not always correspond to protein abundance because of post-transcriptional and translational regulation [22,23]. Therefore, proteomic analysis is essential for directly characterizing drought-responsive changes at the functional protein level. However, the changes in root proteins at the seedling stage of rice during prolonged drought stress remain insufficiently characterized, particularly across different treatment durations. In this study, PEG was used to characterize the dose- and time-dependent drought stress response mechanism in rice seedlings. Since the root is the primary site for drought stress detection, proteins were extracted from rice roots after morphological assessment. To explore the drought stress mechanism of rice, nano-liquid chromatography (LC) combined with mass spectrometry (MS)-based proteomics was performed. Proteomic results were subsequently confirmed by immunoblot, polymerase chain reaction (PCR), and metabolite analyses.

## 2. Results

### 2.1. Morphological Analysis of Rice Under PEG Treatment

**Figure 1:**
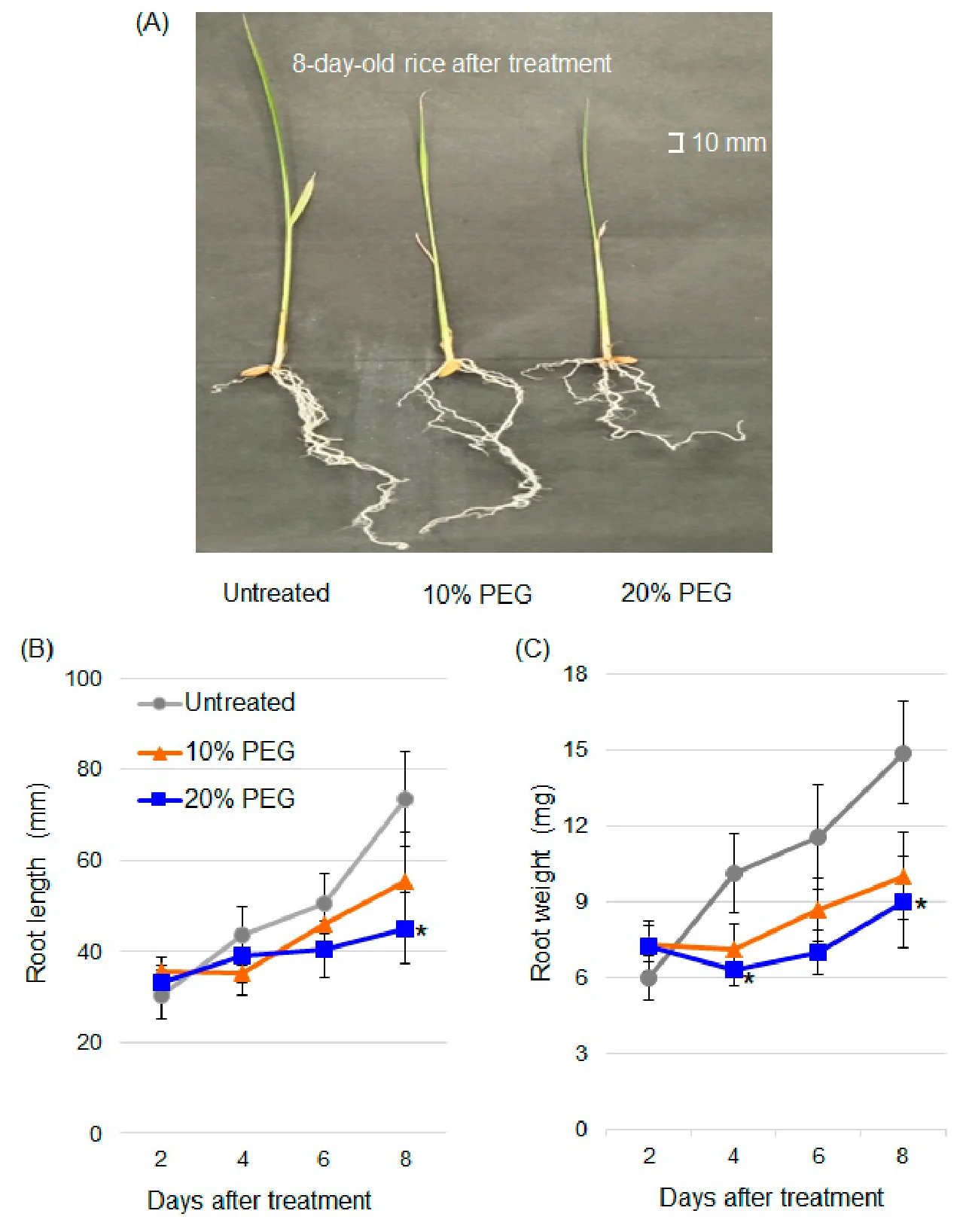
Time- and dose-dependent morphological analysis of rice roots under PEG treatment. Rice soaked for 4 days was sown and 9-day-old seedlings were treated with or without PEG. The bar indicates 10 mm in the picture (**A**). As morphological parameters, main root length (**B**) and total root fresh weight (**C**) were measured at 2, 4, 6, and 8 days after treatment. Gray-, orange-, and blue-colored lines represent untreated, 10% PEG, and 20% PEG, respectively. The data are presented as mean ± SD from 3 independent biological replicates. Student’s *t*-test was utilized to indicate the significant difference between the indicated PEG treatment and the untreated group at the same time point through asterisks (*, *p* < 0.05).

To investigate the effect of PEG treatment on rice roots, morphological analysis was performed. Morphological assessments were conducted at 2, 4, 6, and 8 days following 10 and 20% PEG treatment (Figure S1). Main root length and total root fresh weight were measured as morphological parameters. Primary root length and total root fresh weight increased over time in all treatment groups. During the treatment period, 10% PEG did not significantly affect either parameter. In contrast, prolonged exposure to 20% PEG inhibited rice root growth, significantly reducing both main root length and total root fresh weight after 8 days of treatment. Under 20% PEG, total root fresh weight decreased at 4 days and partially recovered at 6 days, indicating a non-monotonic response (Figure 1). Based on morphological results, rice roots treated with 20% PEG were employed for proteomic analysis and collected at 0, 4, and 8 days of PEG treatment. To evaluate early and prolonged responses to PEG treatment, 4 and 8 days were selected for the following experiments.

### 2.2. Identification and Functional Investigation of Proteins in Rice Under PEG Treatment

**Figure 2:**
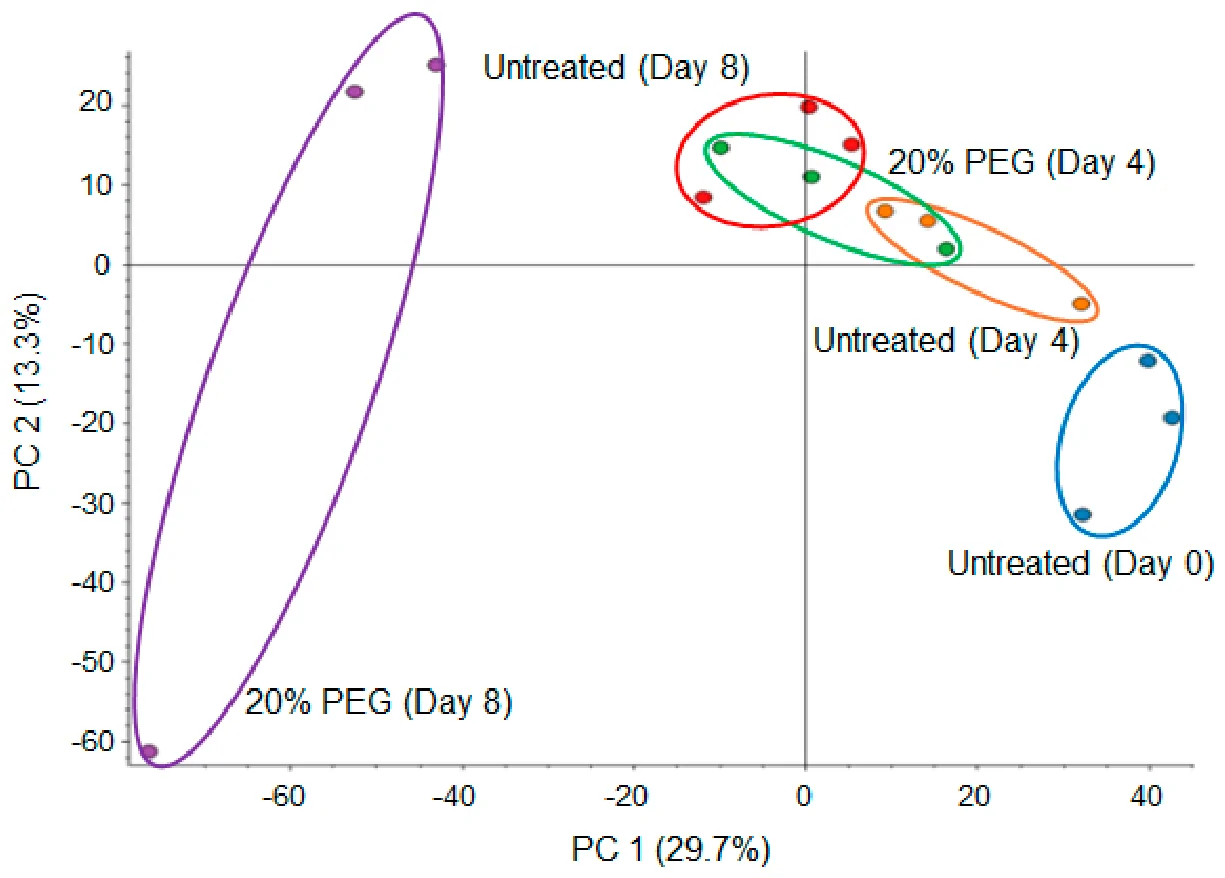
Summary of total proteomic data from 15 samples of rice roots based on principal component analysis. Proteins were extracted from rice roots after 0, 4, and 8 days of PEG treatment. Proteomic analysis was performed with 3 independent biological replicates for each treatment.

To investigate the time-dependent drought stress response mechanism, rice was treated with PEG, and gel-free/label-free proteomics was conducted using roots. Proteins were extracted from rice roots at 0, 4, and 8 days of PEG treatment. Extracted proteins were enriched, reduced, alkylated, digested, and subsequently analyzed by nanoLC-MS/MS and a total of 4637 proteins were identified. The relative protein abundance in PEG-treated rice roots was compared with that of untreated. Principal component analysis was conducted using all 15 samples from five experimental groups across 0, 4, and 8 days of treatment, revealing distinct protein accumulation patterns associated with PEG-treatment (Figure 2).

**Figure 3:**
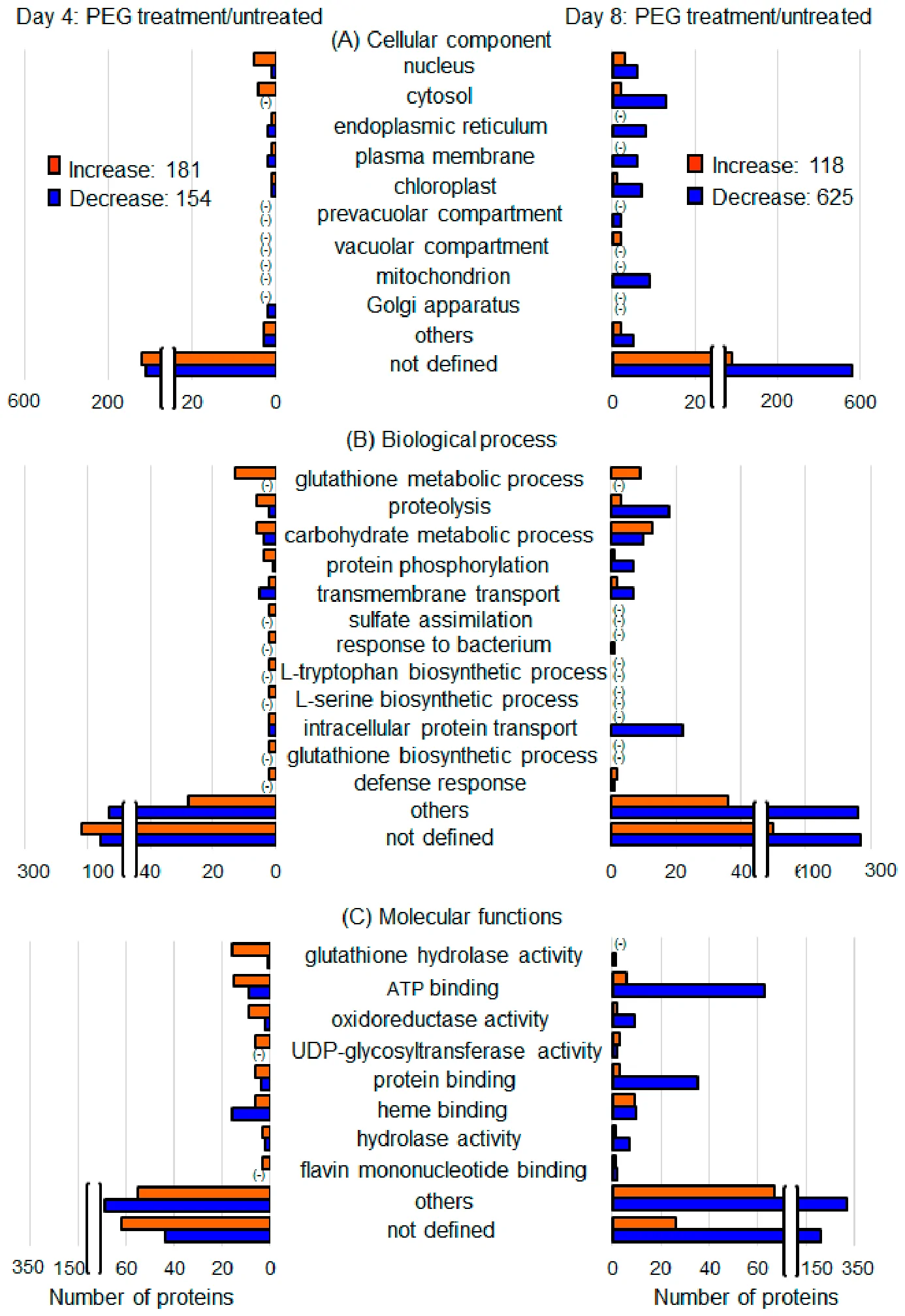
Functional categories of proteins with differential abundance in rice roots under PEG treatment. Proteins extracted from rice roots after 0, 4, and 8 days of PEG treatment underwent enrichment, reduction, alkylation, and enzymatic digestion. After analysis by LC–MS/MS, the relative abundance of proteins from rice roots was compared as follows: day 4: PEG treatment/untreated (Table S1) and day 8: PEG treatment/untreated (Table S2). Functional categories of the changed proteins were determined using Gene Ontology analysis, including cellular component (**A**), biological process (**B**), and molecular function (**C**). Orange and blue columns exhibit increased and decreased proteins, respectively. Others represent Gene Ontology categories not included among the major categories displayed, whereas not defined represents identified proteins for which no corresponding Gene Ontology annotation was assigned.

Proteins identified with at least two matched peptides (matched peptides ≥ 2) and having statistically significant differences in abundance (*p* < 0.05) were considered differentially abundant proteins. At 4 days of PEG treatment, 335 proteins were altered compared with untreated, of which 181 increased and 154 decreased (Table S1). At 8 days of treatment, a total of 743 proteins changed, including 118 increased and 625 decreased compared with untreated (Table S2). Functional classification of the identified proteins was performed using Gene Ontology analysis. The comparisons were made between two groups: 4 days PEG treatment/untreated and 8 days PEG treatment/untreated. To identify biologically meaningful responses to PEG treatment, differentially abundant proteins were first classified based on their abundance patterns at 4 and 8 days. Gene Ontology analysis exhibited an enrichment of proteins annotated as associated with nucleus, protein phosphorylation, and ATP binding (Figure 3). These annotations indicated functional categories represented among the differentially abundant proteins. Proteins related to protein protection, redox/detoxification, and cytoskeletal responses were selected for further investigation based on proteomic results. For selected proteins and transcript level responses, immunoblot and PCR analyses were performed as complementary validation approaches. ATP content was measured as a biochemical indicator of cellular energy status because ATP-binding and energy metabolism-related proteins exhibited different abundance patterns between 4 and 8 days.

### 2.3. Immunoblot Analysis of Proteins Identified by Proteomics in Rice Seedlings Under PEG Treatment

**Figure 4:**
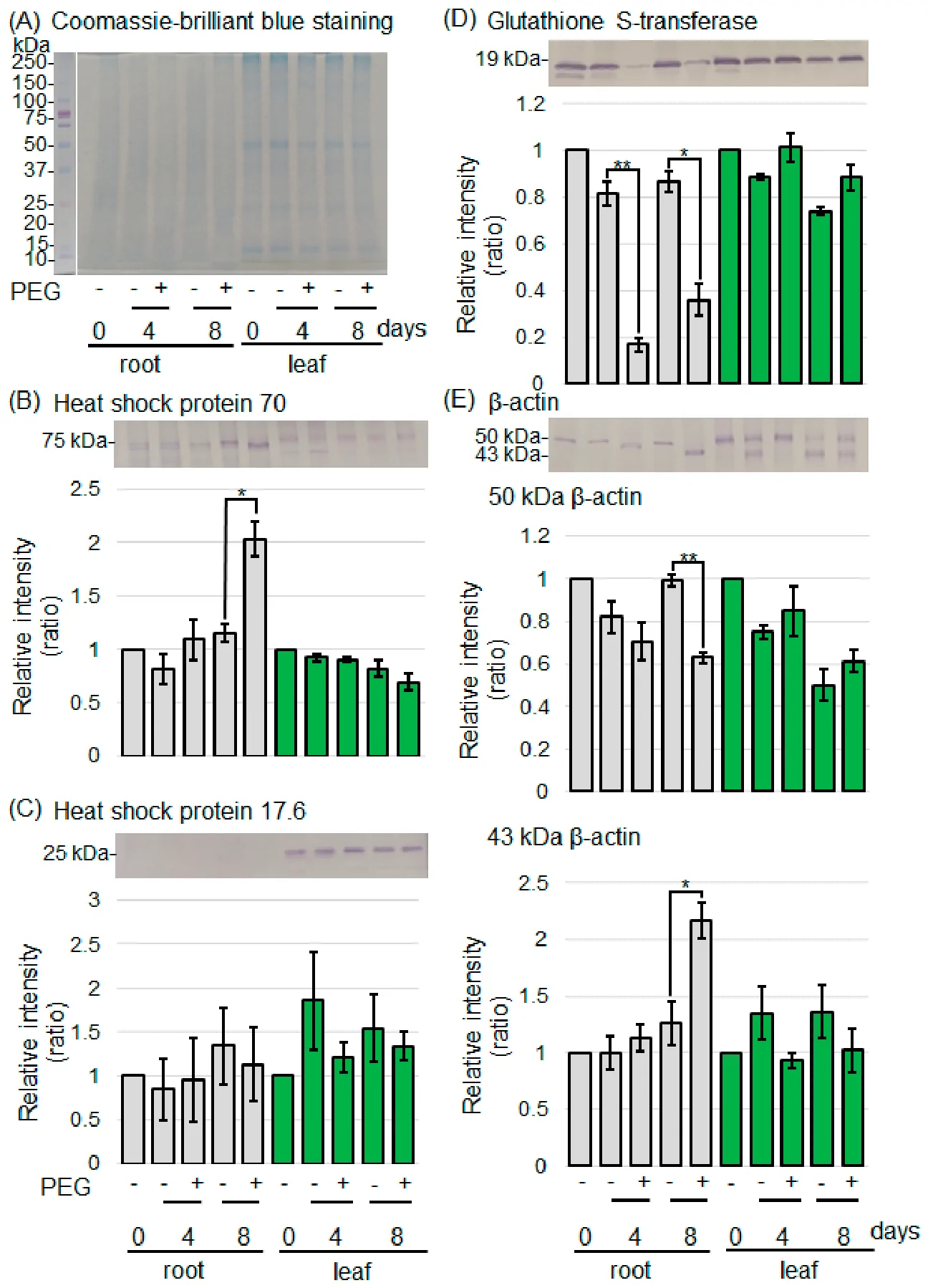
Immunoblot analysis of the proteins involved in rice seedlings under PEG treatment. Proteins extracted from rice roots and leaves after 0, 4, and 8 days of PEG treatment were separated on 10% SDS-polyacrylamide gel by electrophoresis and stained with Coomassie brilliant blue (**A**). The proteins transferred onto membranes were cross-reacted with anti- heat shock protein (HSP) 70 (**B**), HSP17.6 (**C**), glutathione S-transferase (**D**), and β-actin (**E**) antibodies. Gray- and green-colored bars represent root and leaf samples, respectively. For β-actin, the upper and lower bar charts in panel E represent the relative intensities of the 50 kDa and 43 kDa β-actin bands, respectively. The integrated densities of the bands were calculated using ImageJ software. The data are presented as mean ± SD from 3 independent biological replicates (Figures S3–S6). Student’s *t*-test was utilized to indicate the significant difference between untreated and PEG treated groups through asterisks (*, *p* < 0.05; **, *p* < 0.01).

To validate selected protein changes identified in proteomic analysis, immunoblot analysis of heat shock protein (HSP) 70, HSP17.6, glutathione S-transferase, and β-actin was performed at different time points during PEG treatment (Figure 4). HSP70 and HSP17.6 were selected to examine HSP family responses related to molecular chaperone activity, glutathione S-transferase to assess redox and detoxification responses associated with reactive oxygen species (ROS), and β-actin to examine cytoskeleton-related responses, based on the differential abundance of related proteins identified in the proteomic dataset. Proteins extracted from roots and leaves of rice at 0, 4, and 8 days of treatment were separated using SDS-polyacrylamide gel electrophoresis and stained with Coomassie brilliant blue, which was used as a loading control (Figure 4A and Figure S2). Proteins transferred onto membranes were cross-reacted with anti-HSP70 (Figure S3), HSP17.6 (Figure S4), glutathione S-transferase (Figure S5), and β-actin (Figure S6) antibodies. The accumulation of HSP70 increased in roots at 8 days with PEG (Figure 4B), but HSP17.6 did not change with PEG (Figure 4C). In contrast, glutathione S-transferase decreased with PEG treatment at 4 and 8 days (Figure 4D), and 50 kDa β-actin decreased and 43 kDa β-actin increased with PEG treatment at 8 days (Figure 4E).

### 2.4. PCR Analysis of Genes Encoding Proteins Identified by Proteomics in Rice Seedlings Under PEG Treatment

**Figure 5:**
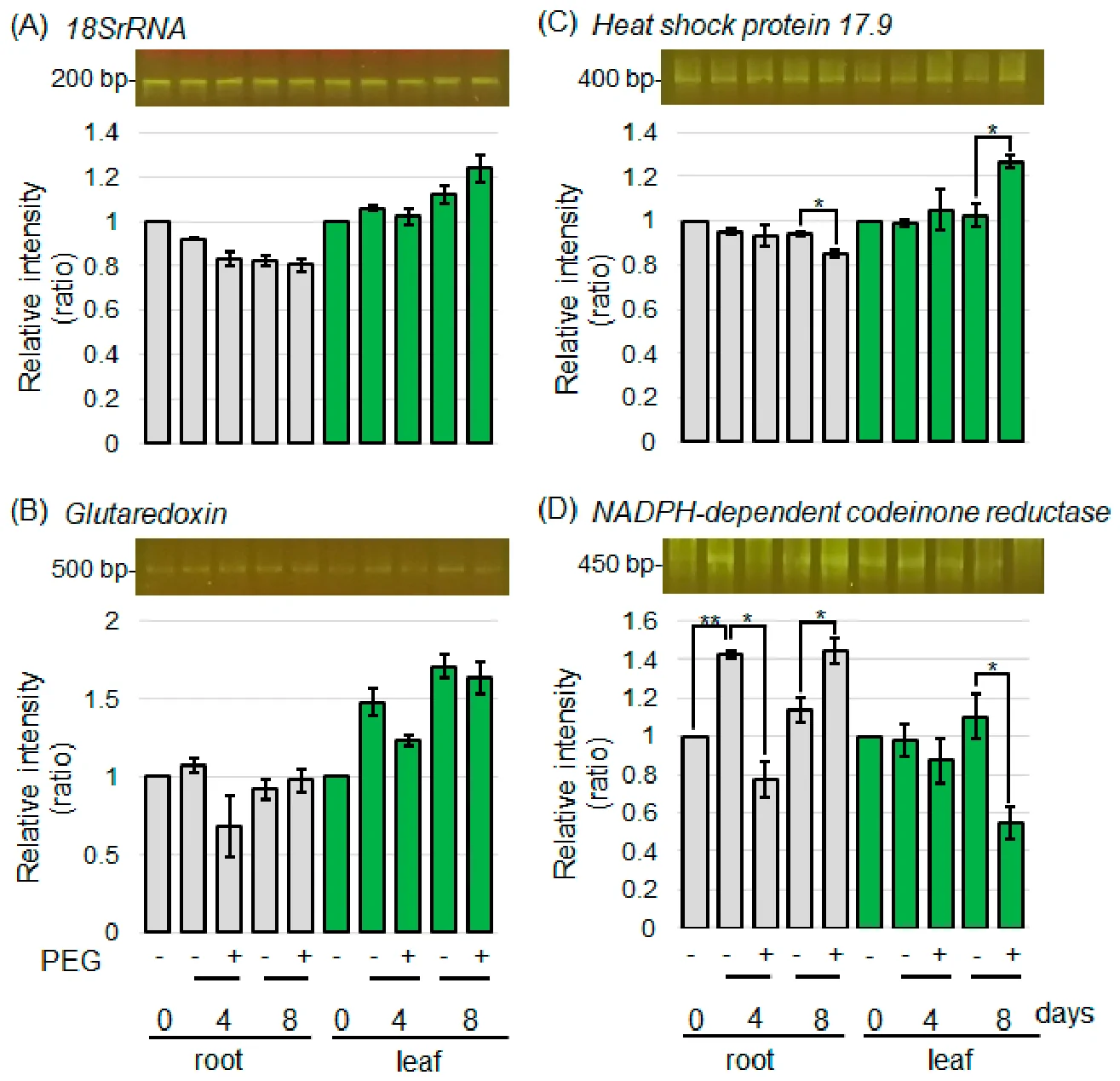
PCR analysis of gene-encoding glutaredoxin, HSP17.9, and NADPH-dependent codeinone reductase proteins in rice seedlings under PEG treatment. After isolating RNAs from rice roots and leaves treated with PEG for 0, 4, and 8 days, *18SrRNA* (**A**)*, glutaredoxin* (**B**), *HSP17.9* (**C**), and *NADPH-dependent codeinone reductase* (**D**)-specific oligonucleotides were amplified using PCR. *18SrRNA* was used as an internal control. Gray- and green-colored bars represent root and leaf samples, respectively. After agarose gel electrophoresis, the integrated densities of bands were calculated using ImageJ software. The data are presented as mean ± SD from 3 independent biological replicates (Figures S7–S10). Student’s *t*-test was utilized to indicate the significant difference between untreated and PEG treated groups through asterisks (*, *p* < 0.05; **, *p* < 0.01).

To better uncover the transcript-level responses of selected proteome-derived candidates, the expression levels of genes encoding the corresponding proteins were analyzed at different time points during PEG treatment using the PCR technique. PCR targets were selected from significantly altered proteins identified by proteomic analysis. Total RNAs isolated from the roots and leaves of rice at 0, 4, and 8 days of treatment were used to synthesize cDNA. PCR analysis was performed for the gene expression of *18SrRNA, glutaredoxin*, *HSP17.9*, and *NADPH-dependent codeinone reductase* (Figures S7–S10). The expression pattern of *18SrRNA* was used as an internal control, which did not change across days and treatment (Figure 5A). *Glutaredoxin*, *HSP17.9*, and *NADPH-dependent codeinone reductase* were selected based on their corresponding proteins changed in the proteomic analysis and were related to redox regulation, protein protection, and reactive carbonyl species detoxification, respectively. The expression level of *glutaredoxin* did not change across days and treatment (Figure 5B). The gene expression level of *HSP17.9* was downregulated in roots and upregulated in leaves by PEG treatment for 8 days (Figure 5C). However, *NADPH-dependent codeinone reductase* was upregulated in roots and downregulated in leaves by PEG treatment for 8 days (Figure 5D).

### 2.5. ATP Content in Rice Seedlings Under PEG Treatment

**Figure 6:**
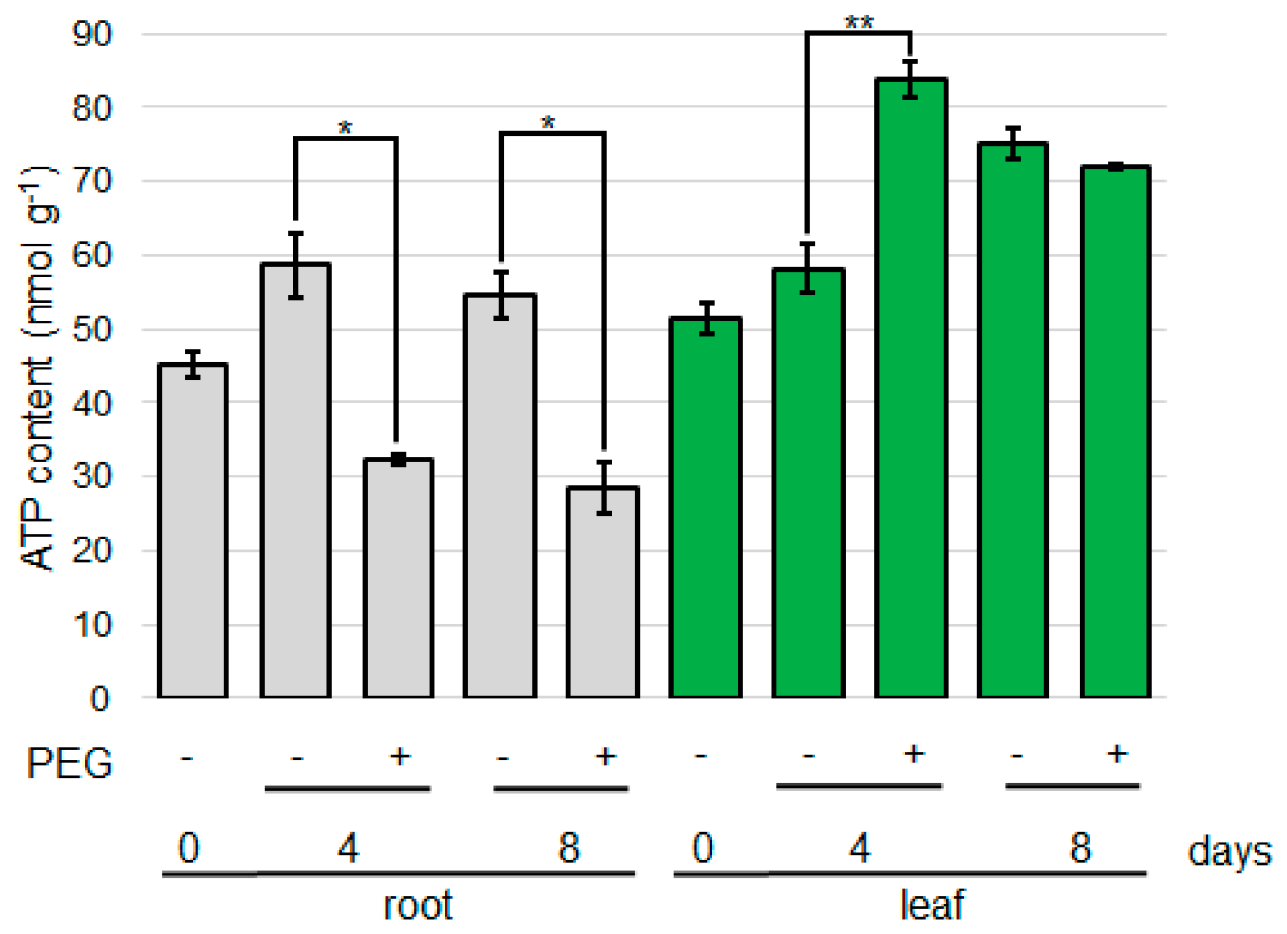
ATP content per unit fresh weight in rice seedlings under PEG treatment. ATP content was measured in roots and leaves at 0, 4, and 8 days after PEG treatment and expressed as nmol ATP g^−1^ fresh weight. Gray- and green-colored bars represent root and leaf samples, respectively. The data are presented as mean ± SD from 3 independent biological replicates. Student’s *t*-test was utilized to indicate the significant difference between untreated and PEG treated groups through asterisks (*, *p* < 0.05; **, *p* < 0.01).

To investigate the energy-related changes suggested by the proteomic analysis, particularly the enrichment of proteins associated with ATP binding, ATP content was measured in the roots and leaves of rice seedlings at 0, 4, and 8 days after PEG treatment. The measured ATP content was expressed as nmol ATP g^−1^ fresh weight. In roots, the ATP content decreased with PEG treatment for 4 and 8 days compared with untreated (Figure 6). In contrast, the ATP content increased in leaves with PEG treatment for 4 days. However, there was no significant difference in leaves with PEG treatment for 8 days compared with untreated.

## 3. Discussion

### 3.1. Osmotic Stress Negatively Affects Rice Root Growth

Rice is highly susceptible to drought stress, and the roots, shoots, and leaves respond differently depending on the growth stage, stress severity, and environmental conditions [8]. Primary root length mainly reflects axial elongation, whereas total root fresh weight reflects biomass influenced by tissue water status, root volume, diameter, and lateral root growth; therefore, these parameters may respond differently under stress [24,25]. PEG is widely used to simulate drought stress by reducing the osmotic potential of the growth medium and limiting water availability to the plant in a controlled way [26]. However, PEG-induced osmotic stress does not fully reproduce natural soil drought because it differs from soil drying and can alter root water uptake, root anatomy, and root architecture [18,27]. PEG treatment reduced plant biomass in sesame [28], passion fruit [26], and triticale [29], with greater reductions at higher PEG concentrations. Seedling height and shoot biomass of maize were reduced by 20% PEG [30]. The root length of Tartary buckwheat was shortened by 20% PEG treatment compared with untreated and 10% PEG treatment [31]. In this study, prolonged exposure to 20% PEG significantly reduced main root length and total root fresh weight after 8 days, whereas 10% PEG did not produce a significant effect (Figure 1). In addition, at 4 days, total root fresh weight was significantly lower under 20% PEG treatment, whereas primary root length was not significantly affected. This difference may reflect reduced tissue hydration or biomass accumulation before primary root elongation was significantly inhibited. Together with previous findings, these results suggest that PEG causes osmotic stress, which becomes stronger with increasing PEG concentrations and longer treatment durations in rice seedlings.

### 3.2. Osmotic Stress Alters the Abundance of Proteins Involved in Reactive ROS Detoxification and Reactive Carbonyl Species Metabolism in Rice Roots

Plants detoxify excess ROS through enzymatic antioxidant systems, such as catalase, glutathione peroxidase, guaiacol peroxidase, glutathione S-transferase, and ascorbate peroxidase [32]. Glutathione S-transferases are part of a large conserved enzyme family, which uses glutathione to detoxify reactive and electrophilic compounds in plant cells [33]. Knockout of *OsGSTU17* impaired rice drought tolerance by increasing oxidative stress, suppressing antioxidant defense system, and regulating drought-responsive gene expression [32]. Overexpression of *OsGSTU30* in *Arabidopsis* improved drought and heavy metal [Cr(vi)] stresses tolerance. Overexpression of *CsGSTU8* in *Arabidopsis* enhanced drought tolerance by scavenging excess amounts of ROS [34]. Overexpression of *PtGSTF1* in poplar enhanced biomass production and salt tolerance, through increased xylem cell number and size, optimized Na^+^/K^+^ homeostasis, and improved scavenging of ROS in transgenic plants [35]. In this study, among the proteins involved in ROS scavenging, glutathione S-transferase decreased in response to PEG treatment (Figure 4), suggesting that drought stress may alter glutathione S-transferase-related detoxification processes in rice roots. Although proteins in the glutathione metabolic process category increased through Gene Ontology analysis, this category includes several glutathione-related proteins and does not represent the response of a single protein or enzyme family. Glutathione S-transferases comprise a large multigene family with multiple isoforms, which may participate in the metabolism of several electrophilic substrates [36]. This result indicates a change in glutathione S-transferase-associated processes rather than a definitive reduction in total ROS detoxification capacity.

NADPH-dependent codeinone reductase belongs to the aldo–keto reductase superfamily and catalyzes the NADPH-dependent reduction of carbonyl groups, thereby contributing to the detoxification of reactive carbonyl species in plants [37,38,39]. Under abiotic stresses, excess ROS attack membrane lipids, producing lipid peroxides, which decompose into reactive aldehydes and ketones, including acrolein, 4-hydroxy-2-nonenal, malondialdehyde, and related α,β-unsaturated carbonyls [40,41]. JcAKR was identified as an aldo–keto reductase from *Jatropha curcas*, which detoxified methylglyoxal and increased tolerance to PEG, salinity, and methylglyoxal when expressed in microbial cells [42]. Overexpression of *aldo–keto reductase 1* in rice and tobacco reduced the accumulation of reactive cytotoxic carbonyls and glycation products, which improved seed viability and seedling vigor [43]. In this study, *NADPH-dependent codeinone reductase* was upregulated in roots by PEG for 8 days (Figure 5). This result, together with previous findings, suggests that drought stress may enhance a reactive carbonyl metabolism-related response in rice roots.

### 3.3. Osmotic Stress Induces Chaperone-Mediated Protein Protection in Rice Roots

HSPs are molecular chaperones, which protect cellular proteins from stress-induced misfolding, aggregation, and loss of function [44]. HSP70 proteins are ATP-dependent molecular chaperones that maintain protein homeostasis by promoting proper protein folding, preventing aggregation, facilitating protein translocation, and targeting damaged proteins for degradation [45]. Small HSPs are ATP-independent molecular chaperones that bind partially unfolded proteins and prevent their aggregation, providing first line defense against stress [46]. In rice, 9 *HSP* genes were upregulated in response to heat, PEG, and ABA treatments, including genes encoding HSP70 and small HSPs [47]. Overexpression of *PpHSP70* improved both high-temperature and drought tolerance in rice [48]. Silencing of *HSP70* caused severe growth inhibition, membrane damage, reduced water content, pigment accumulation, and antioxidant activity in tomato under drought stress [49]. Salt stress upregulated *Hsp17.8*, *HSP26.3*, *HSP70*, and *HSP101* in durum wheat [50]. Overexpression of *SlHSP17.3* improved germination, root growth, chlorophyll retention, and photosynthetic performance, while reducing ROS and membrane injury during salt stress in tomato [51]. Several garlic small *HSP* genes were induced by heat and salinity, indicating that individual small HSPs may function as core stress-responsive genes [52]. In this study, HSP70 increased with PEG treatment for 8 days, whereas HSP17.6 remained unchanged across days and treatment (Figure 4). Furthermore, the gene expression level of *HSP17.9* was downregulated in roots by PEG treatment for 8 days (Figure 5). The present results, together with previous findings, suggest that small HSP-mediated protein protection may be limited, whereas HSP70 may provide ATP-dependent, chaperone-mediated protein protection during prolonged drought stress in rice roots. The abundance of HSP17.6 protein was examined by immunoblot analysis, whereas the expression of *HSP17.9* transcript was examined by semi-quantitative PCR analysis. Because these analyses evaluated different small HSPs, their results were not directly comparable. In addition, gene expression and protein abundance may differ because of post-transcriptional and post-translational regulation [22]. One limitation of the present study is that gene expression was evaluated using semi-quantitative RT-PCR with 18S rRNA as the reference transcript. Although the observed expression patterns were reproducible and consistent with other experimental findings, validation using RT-qPCR and the use of additional stable reference genes would provide a more accurate quantitative assessment of transcript levels and should be addressed in future studies.

### 3.4. Osmotic Stress Alters β-Actin Abundance Patterns in Rice Roots

The plant cytoskeleton comprising microtubules and actin filaments is a highly dynamic cellular component, which continuously remodels in response to internal and external stimuli, contributing to cellular adaptation and the maintenance of plant growth [53]. Cytoskeleton-associated proteins serve as critical regulators of actin cytoskeletal dynamics, coordinating its remodeling in response to environmental stresses [54]. PEG-induced osmotic stress increased actin and actin-1-like proteins in rice roots [55]. Salt stress, which causes osmotic stress, upregulated *actin-7* in rice seedlings [56]. Salt stress reduced specific actin isoforms and caused actin network depletion in *Arabidopsis* roots [57]. A label-free proteomic analysis of eight diverse rice genotypes reported an increased abundance of actin-depolymerizing factor 3 under drought treatment in all genotypes, indicating that drought stress was associated with changes in actin regulatory proteins [17]. In rice aleurone layers, actin microfilament changes were associated with OsVPE3-mediated programmed cell death [58]. In this study, PEG treatment for 8 days altered the β-actin immunoblot pattern, with a decrease in the approximately 50-kDa band and an increase in the approximately 43-kDa band. These changes may reflect an altered abundance of actin-related proteins, isoform-specific responses, proteolytic processing, or stress-related changes in protein stability.

### 3.5. Osmotic Stress Impairs Cellular Energy Status in Rice Roots

ATP is the central energy-carrying molecule that couples energy-releasing reactions to energy-demanding processes, including ion transport, biosynthesis, gene expression, protein turnover, cell expansion, and stress acclimation [59,60]. Drought stress decreased the total ATP content in rice, where sulfide pretreatment restored the ATP content toward the control level [61]. Low salinity enhanced the root ATP content, but higher salinity reduced the root ATP content in ajowan [62]. Under heat stress, the heat-tolerant rice genotype had a higher grain ATP content and higher ATPase activity than the sensitive genotype, which improved the energy status of rice [63]. After 45 °C heat stress, the *ett1* mutants improved cellular energy homeostasis by maintaining a higher ATP content than wild-type rice seedlings [64]. PEG treatment altered tissue water content and fresh mass [65]. In this study, ATP content per unit fresh weight decreased in rice roots after 4 and 8 days of PEG treatment (Figure 6). The present result, together with previous findings, suggests that PEG treatment was associated with a reduced ATP content per unit fresh weight; however, this change may reflect changes in the ATP content, tissue hydration, or both. Furthermore, the increase in HSP70 and decrease in the ATP content in roots, with PEG treatment (Figure 4), suggests that prolonged PEG treatment increased the requirement for ATP-dependent protein protection under limited cellular energy availability.

## 4. Materials and Methods

### 4.1. Plant Material and Treatment

Seeds of rice (*Oryza sativa* L. cv. Koshihikari) were soaked in 150 mL of water for 4 days. After soaking for 4 days, seeds were sown in seedling case with 300 mL of silica sand and nurtured in a growth chamber at 25 °C with 70% relative humidity and white fluorescent light (12 h of light at 200 µmol m^−2^ s^−1^ and 12 h of dark photoperiod). After sowing for 9 days, seedlings were separated into 3 groups, including an untreated control and 2 groups treated with 10 and 20% PEG-6000 (Fujifilm Wako Pure Chemical, Osaka, Japan). The main root length and total root fresh weight were measured at 2, 4, 6, and 8 days of treatment. Based on morphological analysis, 20% PEG was considered for proteomic and other molecular biological analyses. Proteins and RNAs were extracted from rice roots and leaves at 0, 4, and 8 days of treatment. For each experiment, 3 biological replicates were conducted. In each experiment, 20 seeds were sown in a seedling case for each replication of each treatment. To create biological replicas, the seeds were soaked and sown on different days.

### 4.2. Protein Extraction and Concentration Measurement

A portion (500 mg) of samples was ground in 500 µL of extraction buffer consisting of 50 mM Tris-HCl (pH 7.6), 100 mM NaCl, 1% Nonidet-P40, 0.1% SDS, and protease inhibitors (Nacalai Tesque, Kyoto, Japan). The suspension was centrifuged at 16,000× *g* for 10 min at 4 °C. The supernatant was transferred to a new tube and centrifuged again at 16,000× *g* for 10 min at 4 °C. The supernatant was used as the soluble fraction. After mixing and incubation with XL-Bradford protein assay reagent (Integrale, Tokushima, Japan), absorbance was measured at 595 nm and concentrations were determined using standardized protein solutions of bovine serum albumin.

### 4.3. Enrichment, Reduction, Alkylation, and Digestion of Proteins

The quantified protein (100 µg) was adjusted to a final volume of 100 µL. The proteins were concentrated, reduced, alkylated, and digested using previous methods [66]. In brief, 400 µL of methanol, 100 µL of chloroform, and 300 µL of water were mixed with each sample. After mixing and centrifugation at 16,000× *g* for 10 min to achieve phase separation, the upper phase was discarded and 300 µL of methanol was added to the lower phase. After centrifugation at 16,000× *g* for 10 min, the pellet was resuspended in 50 mM ammonium bicarbonate. Proteins were reduced with 50 mM dithiothreitol for 30 min at 56 °C and alkylated with 50 mM iodoacetamide for 30 min at 37 °C in the dark. Following incubation, alkylated proteins were digested with trypsin and lysyl endopeptidase (Fujifilm Wako Pure Chemical) at a 1:100 enzyme/protein ratio for 16 h at 37 °C. Peptides were desalted with MonoSpin C18 Column (GL Sciences, Tokyo, Japan) and acidified with 1% trifluoroacetic acid.

### 4.4. Identification of Proteins Using nanoLC-MS/MS

The nanoLC conditions (EASY-nLC 1000; Thermo Fisher Scientific, San Jose, CA, USA) and MS acquisition (Orbitrap Fusion ETD MS; Thermo Fisher Scientific) are described in the previous study [67]. In brief, the peptides were loaded onto the LC system equipped with a trap column (Acclaim PepMap 100 C18 LC column, 3 µm, 75 µm ID × 20 mm; Thermo Fisher Scientific), equilibrated with 0.1% formic acid, and eluted with a linear acetonitrile gradient (0–35%) in 0.1% formic acid at a flow rate of 300 nL min^−1^. The eluted peptides were loaded and separated on the column (EASY-Spray C18 LC column, 3 µm, 75 µm ID × 150 mm; Thermo Fisher Scientific) with a spray voltage of 2 kV (Ion Transfer Tube temperature: 275 °C). The peptide ions were detected using MS in the data-dependent acquisition mode with the installed Xcalibur software (version 4.7.69.37; Thermo Fisher Scientific). Full-scan mass spectra were acquired in the MS over 375–1500 *m*/*z* with a resolution of 120,000. The most intense precursor ions were picked for collision-induced fragmentation in the linear ion trap with a normalized collision energy of 35%. Dynamic exclusion was employed within 60 s to prevent repetitive selection of peptides.

### 4.5. Analysis of MS Data

The MS/MS data were searched using the MASCOT (version 2.6.2; Matrix Science, London, UK) and SEQUEST HT search algorithms against the IRGSP-1.0_protein_2024-07-12.fasta database (downloaded from The Rice Annotation Project Database (RAP-DB) https://rapdb.dna.naro.go.jp/index.html, accessed by 30 December 2025) using Proteome Discoverer (version 2.4.1.15; Thermo Fisher Scientific). The workflow is outlined in the previous research [68]. In brief, the workflow for both algorithms included spectrum files RC, spectrum selector, MASCOT, SEQUEST HT search nodes, percolator, ptmRS, and Minora Feature Detector nodes. Methionine oxidation was considered as a variable modification, while cysteine carbamidomethylation was regarded as a fixed modification. The MS and MS/MS mass tolerances were set at 10 ppm and 0.6 Da, respectively. Trypsin was specified as a protease, and a maximum of 2 missed cleavages was allowed. The false discovery rate was calculated using target decoy database searches, which was set at 1% for peptide identification.

### 4.6. Differential Analysis of Proteins Using MS Data

Label-free quantification was also performed with Proteome Discoverer 2.4 using precursor ions quantifier nodes. Proteome Discoverer 2.4 was used to conduct principal component analysis. The free software PERSEUS (version 1.6.15.0) [69] was utilized for the differential analysis of the relative abundance of peptides and proteins between samples. The process is detailed in the previous study [68]. In brief, transformed into log2 scale. Each sample included 3 biological replicates, and at least one group needed to contain at least 3 valid values. The median of each sample was subtracted after the abundances were normalized. A normal distribution was used to impute missing data (width = 0.3, down-shift = 1.8). Student’s *t*-test analysis was used to determine significance. A BLAST query against the Gene Ontology database was performed on the sequences of the differentially accumulating proteins.

### 4.7. Immunoblot Analysis

Laemmli sample buffer (Bio-Rad, Hercules, CA, USA), which contained 62.5 mM Tris-HCl (pH 6.8), 1% SDS, 10% glycerol, and 0.005% bromophenol blue along with 50 mM dithiothreitol, was added to protein extracts. Based on the protein concentrations measured with the Bradford method, which is explained in 4.2, the protein extracts were diluted with extraction buffer to 1 μg/μL. Proteins (10 µg) were electrophoretically separated on a 10% SDS-polyacrylamide gel and stained with Coomassie brilliant blue for adjustment of concentration. Proteins were transferred using a semidry transfer blotter onto a polyvinylidene difluoride (PVDF) membrane. Bullet Blocking One reagent (Nacalai Tesque) was employed to block the blotted PVDF membrane for 5 min. Following blocking, the PVDF membrane was cross-reacted with primary antibodies for 30 min. As primary antibodies, anti-HSP70 (StressMarq Biosciences, Victria, Canada), anti-HSP17.6 (Agrisera, Vännäs, Sweden), anti-glutathione S-transferase (Merck, Darmstadt, Germany), and anti-β-actin (Proteintech, Rosemont, IL, USA) were used. The secondary antibody applied was anti-rabbit IgG along with horseradish peroxidase (Bio-Rad). After 30 min of incubation, signals were detected with a solution of 3,3′,5,5′-tetramethylbenzidine (Nacalai Tesque). The integrated density of each target protein band was measured using ImageJ software (version 1.54; National Institutes of Health, Bethesda, MD, USA). Background intensity was measured in an adjacent area and subtracted from the corresponding band intensity to obtain the background-corrected band intensity. Corresponding band intensity was divided with control band intensity to find the ratio.

### 4.8. RNA Extraction, cDNA Synthesis, and PCR Analysis

A portion (500 mg) of samples was ground into a powder using a mortar and pestle after being flash-frozen in liquid nitrogen. Total RNA was extracted using the RNeasy Plant Mini Kit (Qiagen, Venlo, The Netherlands). First-strand cDNA was synthesized from total RNA (1 μg) with the iSuperscript Reverse Transcription Supermix (Bio-Rad). Primers were designed against the specific RAP-DB gene models corresponding to the proteins identified in the proteomic dataset. Primer3Plus (https://www.bioinformatics.nl/cgi-bin/primer3plus/primer3plus.cgi/) [70] was used to construct gene specific primer pairs with an expected amplicon length of 201–500 bp. Protein accession number, primer sequences, and gene symbols are listed in Table S3. PCR analysis was performed using the Emerald Amp PCR Master Mix (Takara, Tokyo, Japan) as follows: at 98 °C for 10 s, at 60 °C for 30 s, and at 72 °C for 30 s, for a total of 30 cycles. The amplified products were separated on 3% agarose gel and stained with the Atlas Clear Sight Gold DNA stain (BioAtlas, Tartu, Estonia). The integrated density of bands was calculated using ImageJ software.

### 4.9. Measurement of ATP Content

A portion (125 mg) of samples was homogenized in 250 μL of the ATP assay buffer and centrifuged at 16,000× *g* for 10 min at 4 °C. For sample deproteinization and neutralization, the supernatant was treated with a Deproteinizing Sample Preparation Kit (Biovision, Milpitas, CA, USA). Extracts (50 μL) were added to 50 μL of a reaction mixture consisting of ATP converter, ATP probe, ATP developer, and ATP assay buffer in an ATP Colorimetric/Fluorometric Assay Kit (Biovision). After mixing and incubation for 30 min at 25 °C in the dark, absorbance was measured at 570 nm and concentrations were determined using standardized ATP solutions as nmol ATP g^−1^ fresh weight.

### 4.10. Statistical Analysis of Data

The statistical significance of data between 2 groups was analyzed by Student’s *t*-test. A *p*-value of less than 0.05 was considered as statistically significant.

## 5. Conclusions

**Figure 7:**
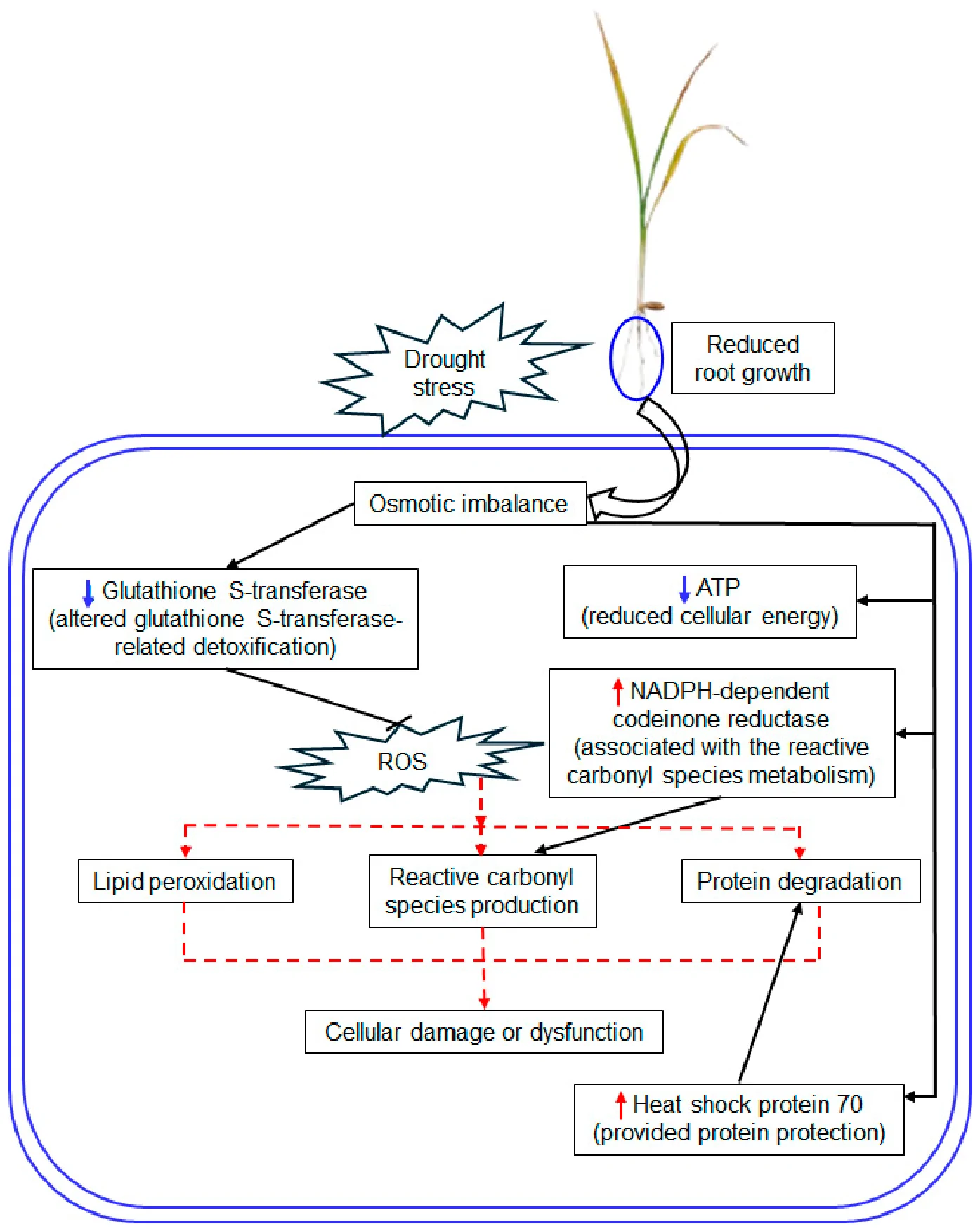
Altered proteins in rice root under PEG-induced osmotic stress compared with untreated. The altered proteins according to the results obtained in the present study were mapped onto a schematic diagram. Blue arrows indicate decreased proteins or metabolites, red arrows indicate increased proteins or metabolites, and red dotted arrows indicate proposed associations among stress-related processes.

Drought stress, primarily through osmotic imbalance, severely limits rice growth and productivity, yet the molecular mechanisms underlying root responses at the seedling stage to prolonged water deficit remain incompletely characterized. To clarify the molecular basis of drought responses in rice at the seedling stage, nanoLC-MS/MS-based proteomic analysis of rice roots under PEG-induced osmotic stress was performed and confirmed by immunoblot, PCR, and metabolite analyses. The altered proteins according to the results obtained in the present study were mapped onto a schematic diagram (Figure 7). The main findings of this study are as follows: (i) prolonged treatment with 20% PEG significantly reduced main root length and total root fresh weight after 8 days; (ii) proteomics identified PEG-associated changes in different rice root proteins between 4 and 8 days, with Gene Ontology annotations related to nuclear components, protein phosphorylation, and ATP binding; (iii) glutathione S-transferase decreased and HSP70 increased in roots with PEG treatment; (iv) the gene expression of *HSP17.9* was downregulated and *NADPH-dependent codeinone reductase* was upregulated in roots by PEG treatment; and (v) ATP content per unit fresh weight declined with PEG. Overall, prolonged PEG-induced osmotic stress suppresses rice root growth by disrupting cellular energy status, altering the abundance of proteins involved in protein protection and glutathione S-transferase-associated detoxification, and modulating the expression of genes associated with reactive carbonyl species metabolism.

## Data Availability

MS data, RAW data, peak lists, and result files of proteomic analysis have been deposited in the ProteomeXchange Consortium [71] via the jPOST [72] partner repository under dataset identifier PXD072795.

## Supplementary Materials

The following supporting information can be downloaded at https://www.mdpi.com/article/10.3390/ijms27198895/s1.

## Abbreviations

The following abbreviations are used in this manuscript:

PEG	Polyethylene glycol
ROS	Reactive oxygen species
LC	Liquid chromatography
MS	Mass spectrometry
PCR	Polymerase chain reaction
