# Supplementary material for: Proteomic and Molecular Biological Analyses of Response Mechanism in Rice (Oryza sativa L. cv. Koshihikari) Under Drought Stress
Source: Int J Mol Sci. 2026 Oct 7;27(19):8895. doi: 10.3390/ijms27198895 (PMC13664928; doi:10.3390/ijms27198895)
Supplement: Supplementary file 1 [file ijms-27-08895-s001.zip › Supplementary Figures.pdf]

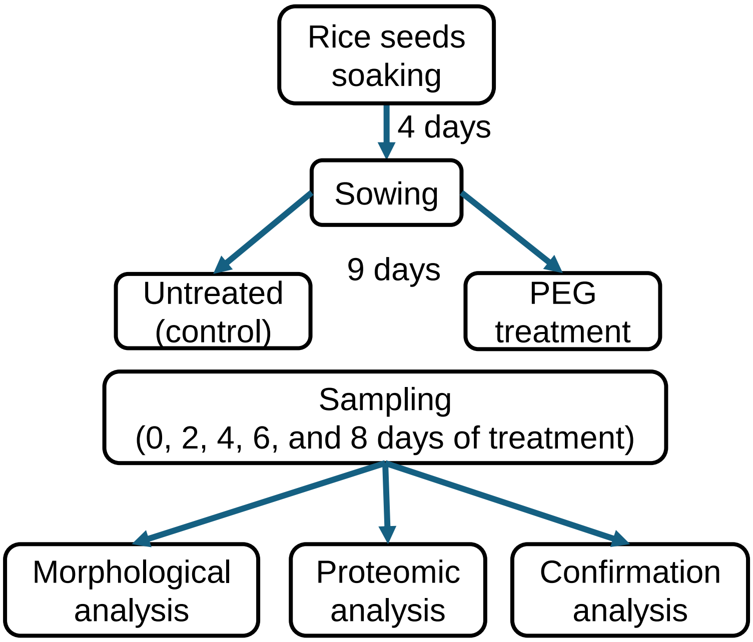

Figure S1. Experimental design for investigating the effects of PEG treatment in rice. Rice soaked for 4 days was sown and 9-day-old seedlings were treated with or without PEG. Rice roots were subjected to morphological and proteomic analyses. To confirm the proteomic analysis result, immunoblot, PCR, and metabolite analyses were performed as confirmation analysis. All experiments were conducted using 3 independent biological replicates.

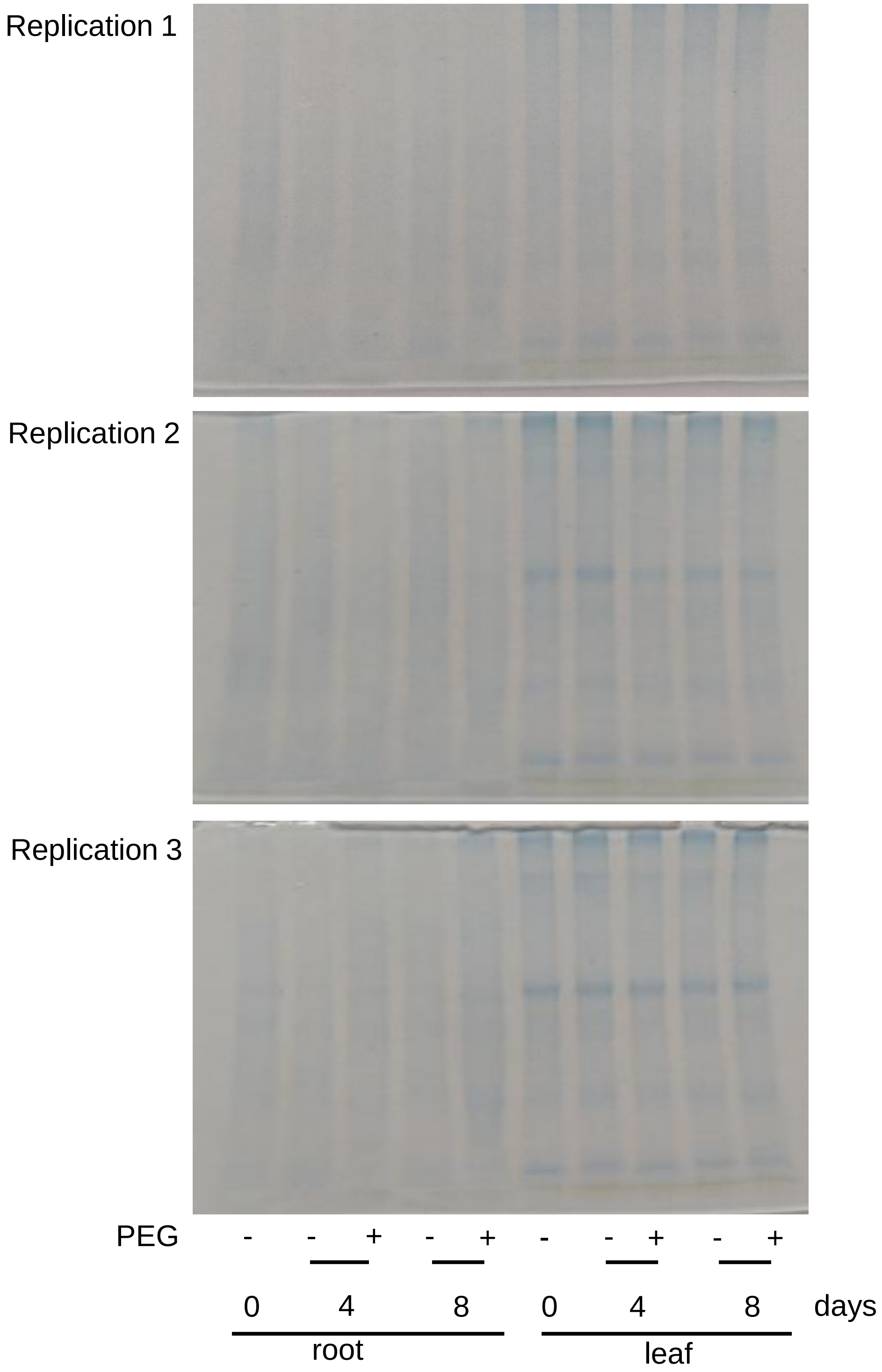

Figure S2. The Coomassie-brilliant blue staining pattern of proteins used for immunoblot analysis, which was used in Figure 4A. Experiments were performed with biologically triplicates for each treatments. Quantified proteins (10 μg) from roots and leaves were separated by electrophoresis on a 10% SDS-polyacrylamide gel. Coomassie-brilliant blue staining was used as a loading control.

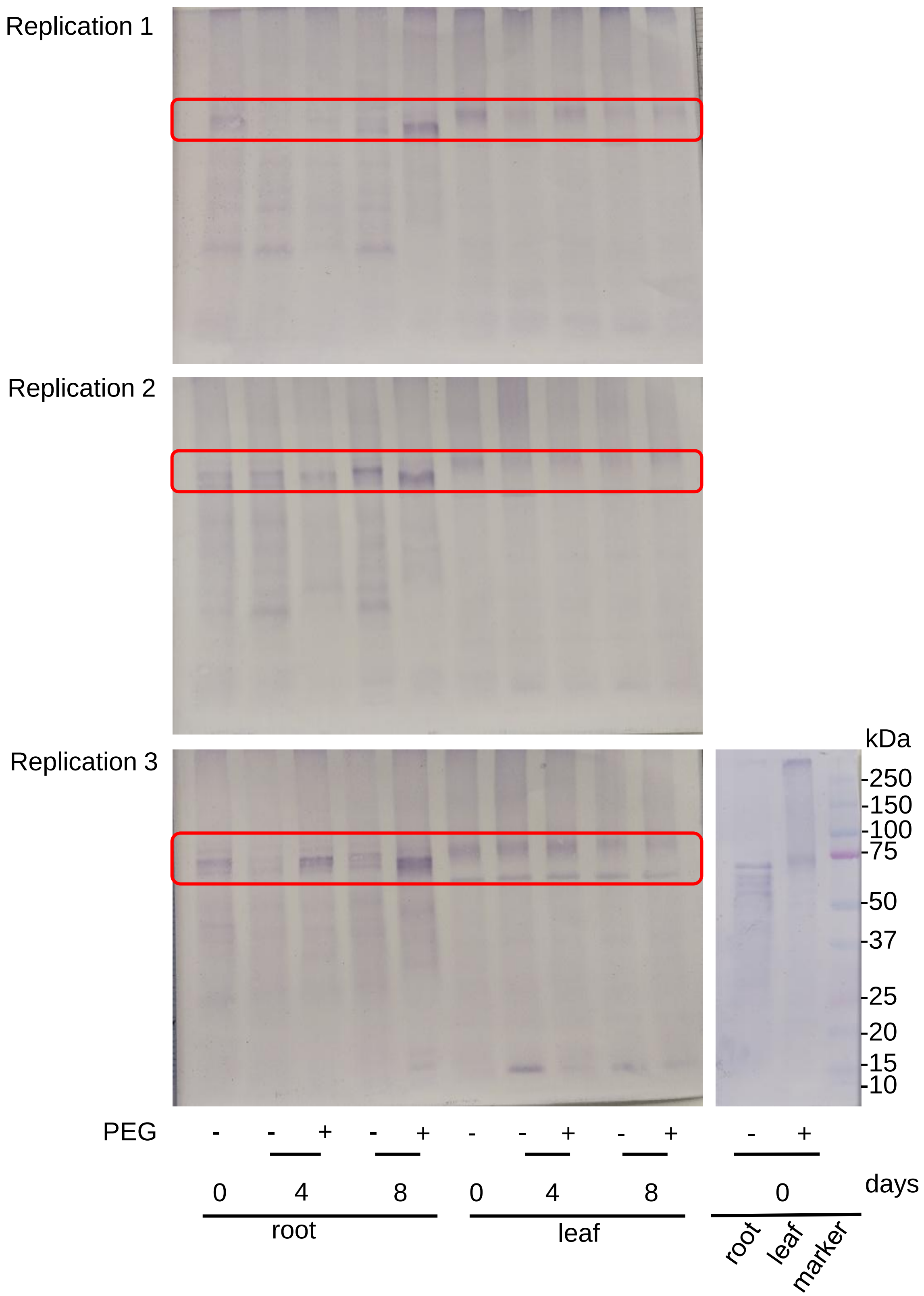

Figure S3. Blots of the entire membrane with anti-heat shock protein 70 antibody, which were used in Figure 4B.

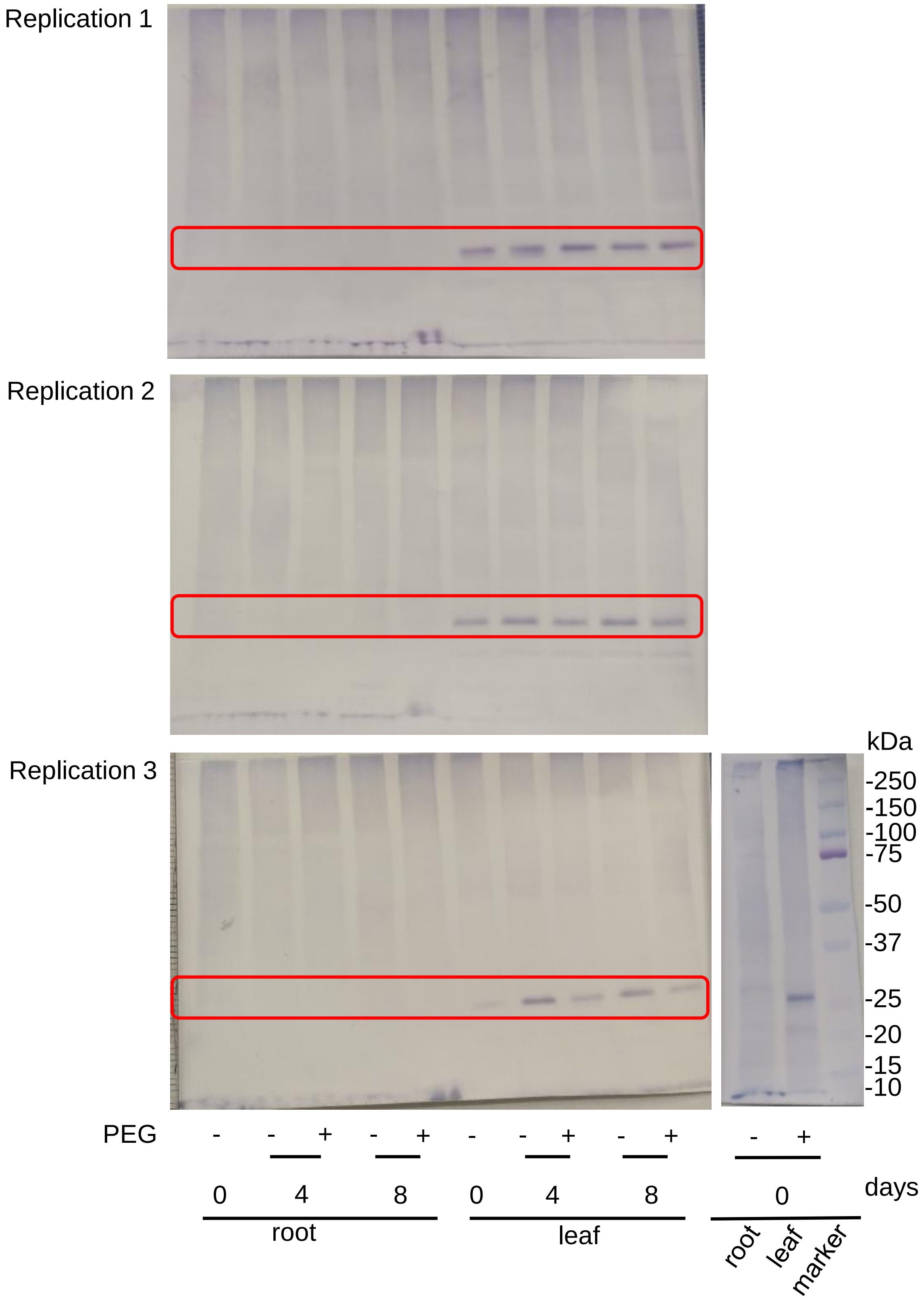

Figure S4. Blots of the entire membrane with anti-heat shock protein 17.6 antibody, which were used in Figure 4C.

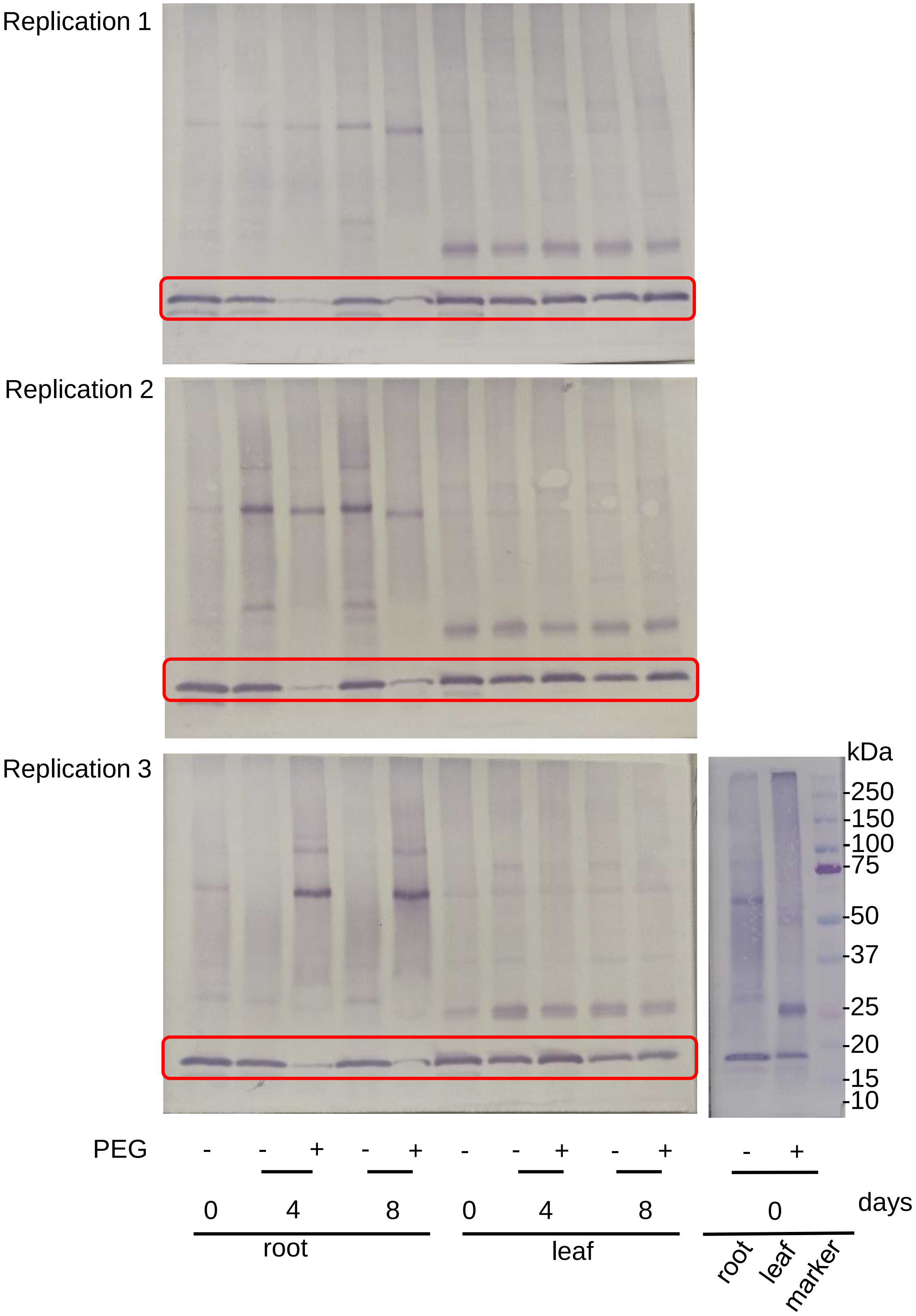

Figure S5. Blots of the entire membrane with anti-glutathione S-transferase antibody, which were used in Figure 4D.

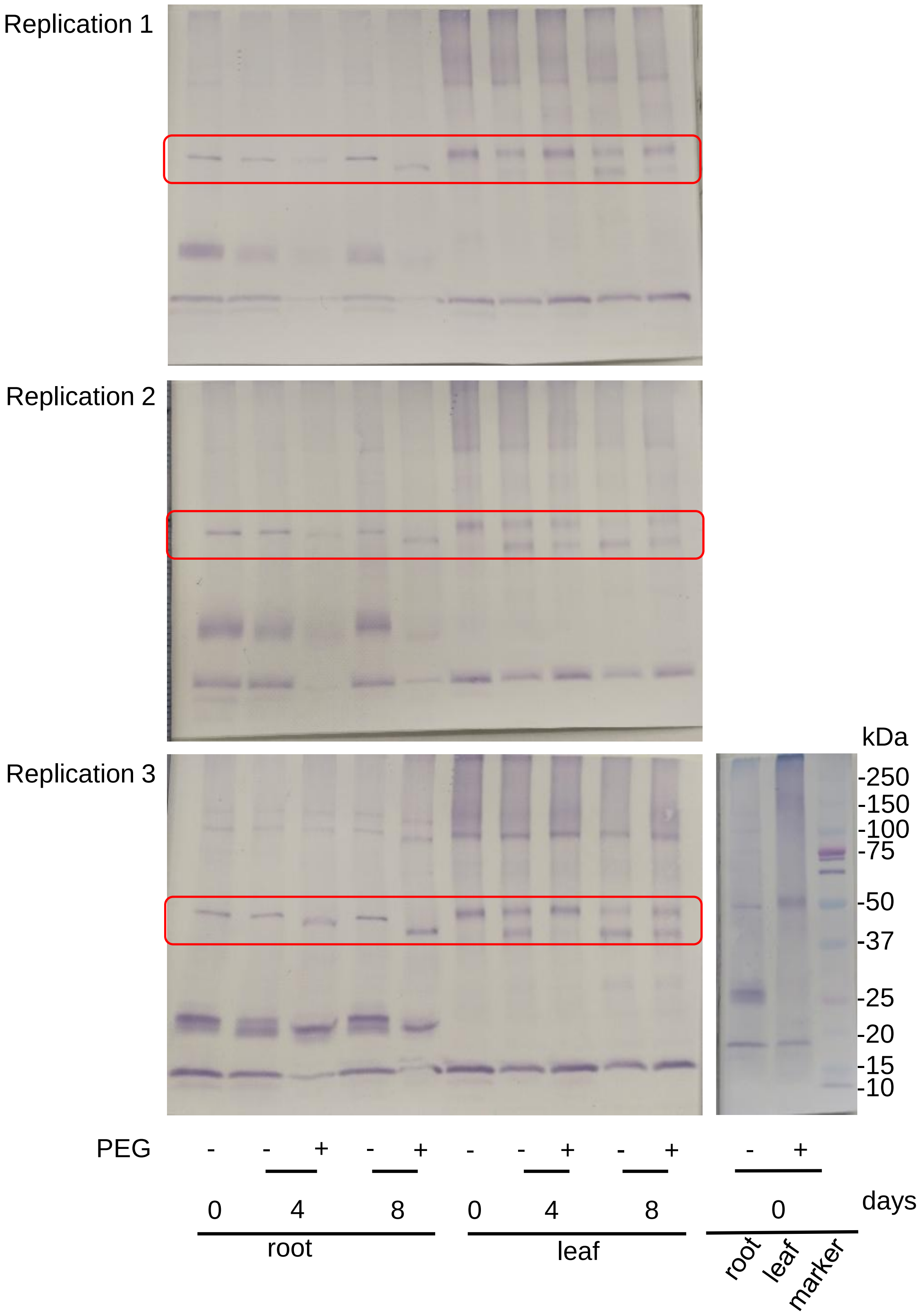

Figure S6. Blots of the entire membrane with anti-β-actin antibody, which were used in Figure 4E for 50 kDa and 43 kDa β-actin.

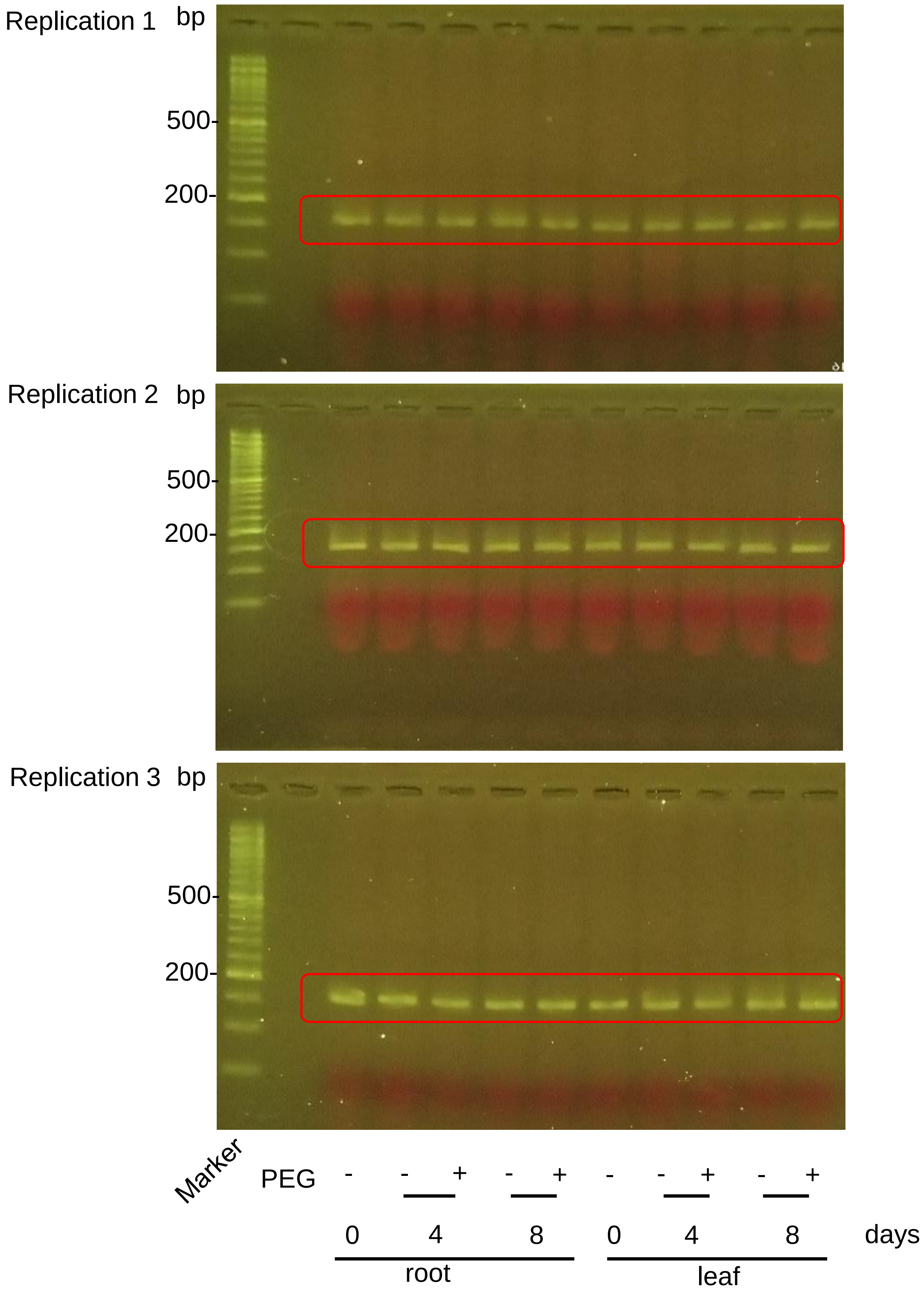

Figure S7. Patterns of agarose gel with *18SrRNA,* which were used in Figure 5A.

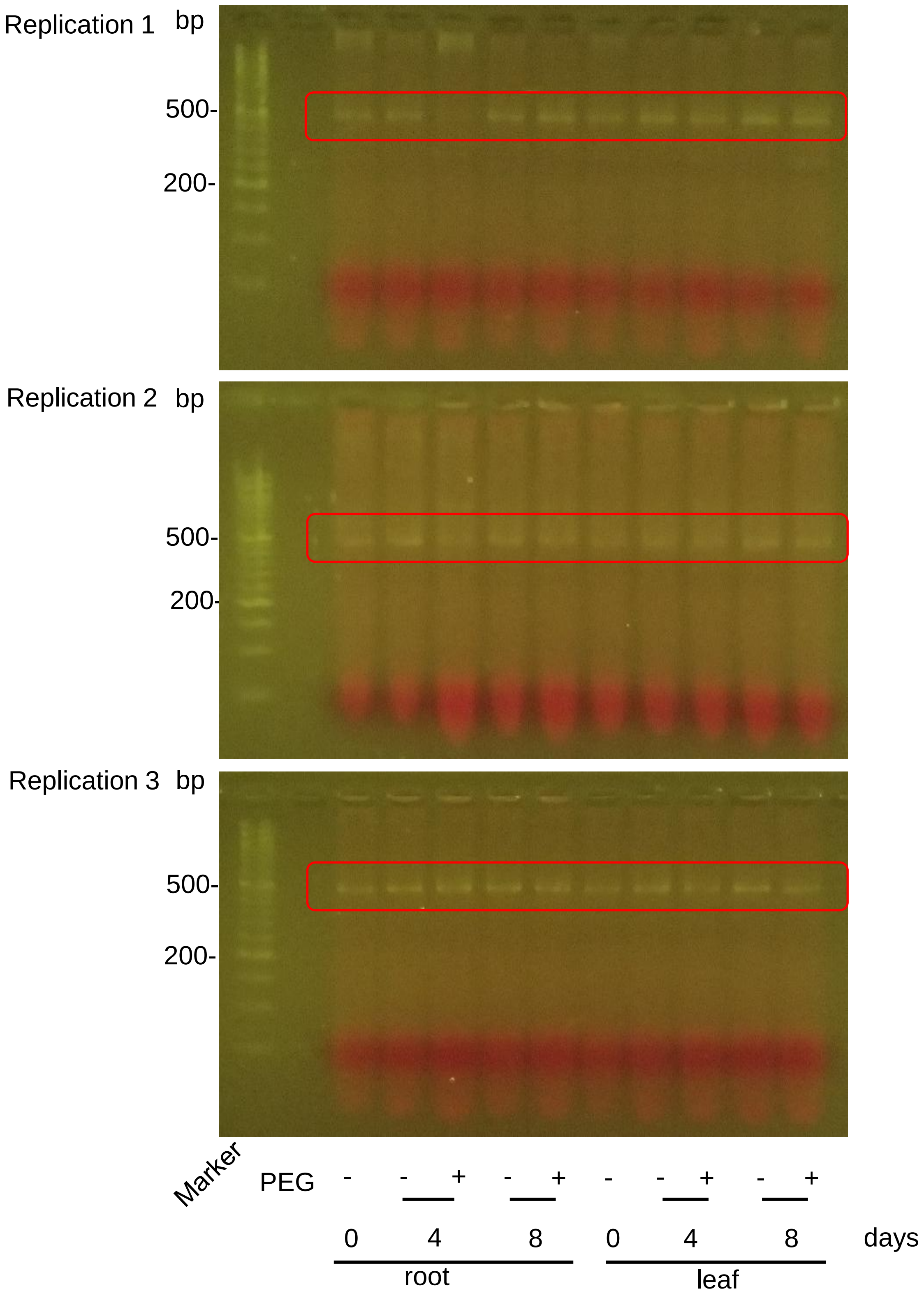

Figure S8. Patterns of agarose gel with *glutaredoxin,* which were used in Figure 5B.

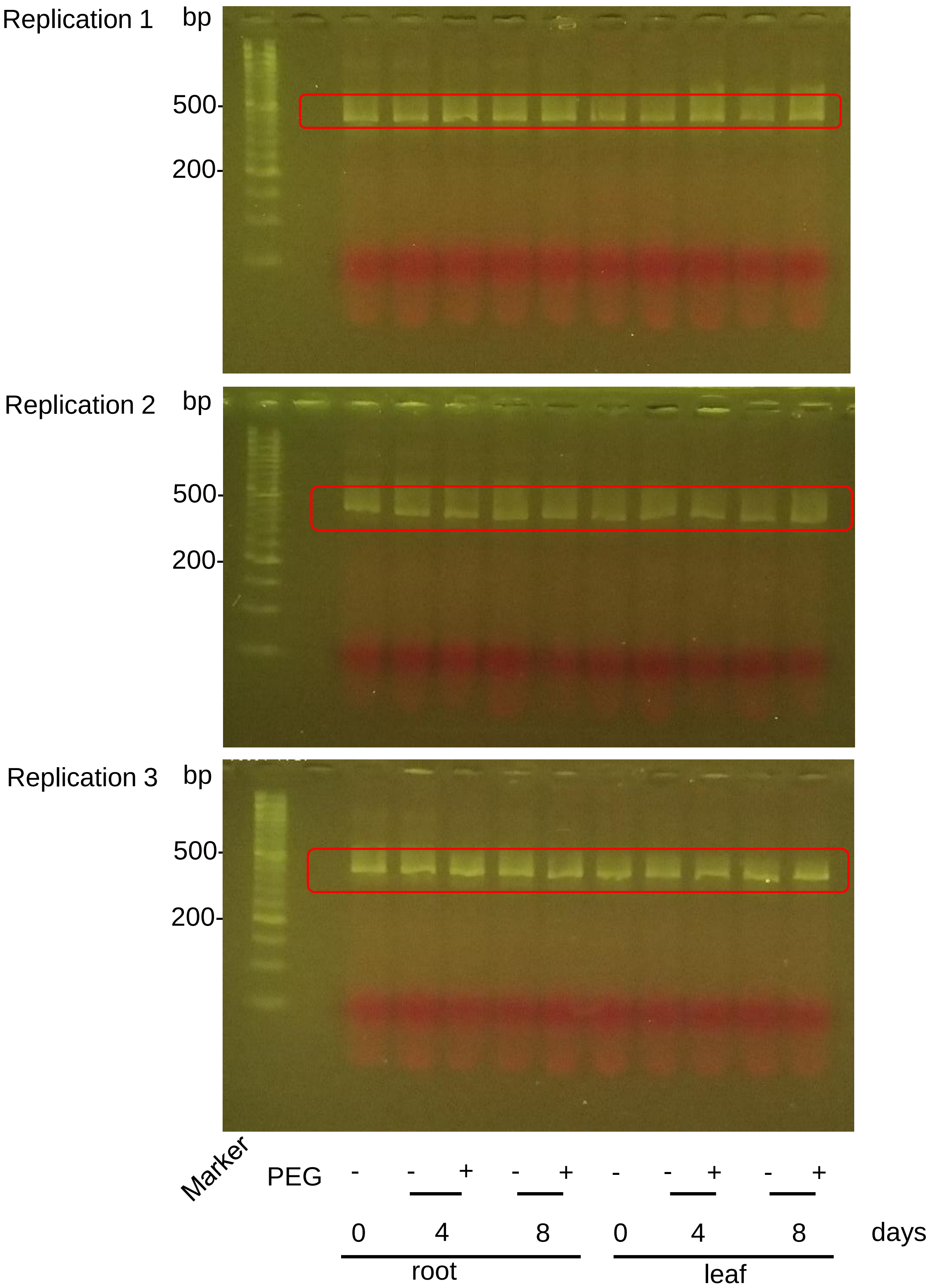

Figure S9. Patterns of agarose gel with *heat shock protein 17.9*, which were used in Figure 5C.

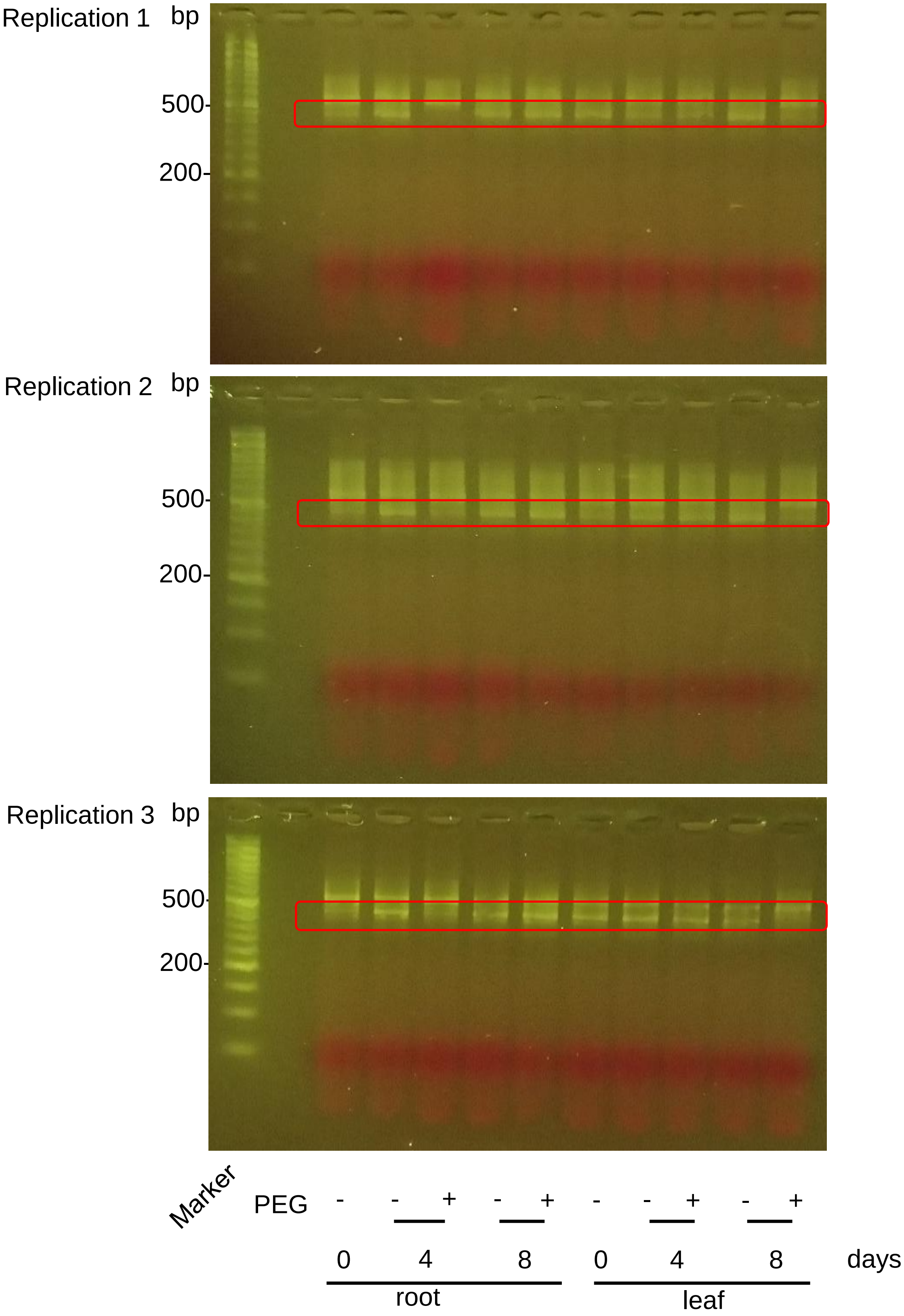

Figure S10. Patterns of agarose gel with *NADPH-dependent codeinone reductase*, which were used in Figure 5D.
